# Caspase-11, a specific sensor for intracellular lipopolysaccharide recognition, mediates the non-canonical inflammatory pathway of pyroptosis

**DOI:** 10.1186/s13578-019-0292-0

**Published:** 2019-03-27

**Authors:** Xiaoli Huang, Yang Feng, Guanqing Xiong, Shona Whyte, Jing Duan, Yucen Yang, Kaiyu Wang, Shiyong Yang, Yi Geng, Yangping Ou, Defang Chen

**Affiliations:** 1 College of Animal Science & Technology, Sichuan Agricultural University, Chengdu, 611130 Sichuan China; 2 Atlantic Veterinary College, University of Prince Edward Island, Charlottetown, C1A 8Z2 PEI Canada; 3 College of Veterinary Medicine, Sichuan Agricultural University, Chengdu, 611130 Sichuan China

**Keywords:** Pyroptosis, Non-canonical inflammatory pathway, Caspase-1, Caspase-11

## Abstract

Pyroptosis, a type of programmed cell death that along with inflammation, is mainly regulated by two main pathways, cysteinyl aspartate specific proteinase (caspase)-1-induced canonical inflammatory pathway and caspase-11-induced non-canonical inflammatory pathway. The non-canonical inflammatory pathway-induced pyroptosis is a unique immune response in response to gram-negative (G^−^) bacteria. It is induced by lipopolysaccharide (LPS) on the surface of G^−^ bacteria. This activates caspase-11 which, in turn, activates a series of downstream proteins eventually forming protein pores on the cell membrane and inducing cell sacrificial processes. Caspase-11 belongs to the caspase family and is an homologous protein of caspase-1. It has the ability to specifically hydrolyze proteins, but it is still unclear how it regulates cell death caused by non-canonical inflammatory pathways. The present study describes a pathway that enables LPS to directly enter the cell and activate caspase-11, and the key role caspase-11 plays in the activation of pyroptosis and inflammation.

## Introduction

Programmed cell death is an orderly phenomenon of cellular self-destruction. It is vital for the development of the organism, metamorphosis, tissue homeostasis and pathogenesis and triggered by both intrinsic and extrinsic factors. Programmed cell death, serves to remove unneeded or potentially harmful cells from the body [1]. At the end of the last century, there were two types of programmed cell death: apoptosis and necroptosis, a programmed form of necrosis [2]. With advances in science and technology, researchers have discovered another process of programmed cell death that is different from apoptosis or necroptosis, namely pyroptosis [3], which occupies an important role as an inflammatory form of programmed cell death in host immunity and essential for controlling microbial pathogens infections. In pyroptosis, caspase-1 is activated and mediates cell death through the processing and release of pro-inflammatory cytokines, interleukin-1β (IL-1β) and IL-18 by rapid rupture of the cell plasma membrane. This inflammatory response is fatal and results in cell death [4, 5]. Two gene pathways, cysteinyl aspartate specific proteinase (caspase)-1 induced canonical inflammatory pathway and caspase-11 induced non-canonical inflammatory pathway, have been suggested to regulate pyroptosis [6]. The canonical inflammatory pathway is stimulated by a range of microbial infections and non-infectious stimuli [7], whereas the non-canonical inflammatory pathway is activated by intracellular LPS derived from gram-negative (G^−^) bacteria [8]. However, most studies have focused on the validation of the canonical inflammatory pathway and there are limited comprehensive reviews of the regulation of non-canonical inflammatory pathways [9, 10]. This review aims to summarize the current research on non-canonical inflammatory pathways, elaborate on the mechanism, and summarize the similarities and differences between the two pathways.

## Discovery of pyroptosis

In 1972, Kerr and colleagues observed that ‘shrinkage necrosis’ had a regulatory role in the morphological patterns of cell death in animal cell populations. They renamed this processed apoptosis [11, 12]. Apoptosis involves condensation of the nucleus and cytoplasm, cellular fragmentation into membrane bound protuberances which form on the cell surface. In 1988, Laster discovered a phenomenon of fragmentation of cell membranes and release of contents in the different types of cells induced by tumor necrosis factor (TNF)-α in the process of necrosis [13]. Since then, many studies have found gene regulatory networks in these so-called ‘necrosis’. In 2005, Degterev proposed the concept of “necroptosis” as distinct from conventional necrosis [14], which was mainly characterized by cell swelling and roundness, cell organelle expansion and cell membrane distension and rupture [15]. When the cell membrane was broken, the cytoplasm would release its contents, which contains proinflammatory cytokines, into the extracellular space and would trigger an inflammatory response [16].

Early in 1996, a different process of cell death was emerging. Chen et al. microinjected *Shigella flexneri* into macrophages and observed that *S. flexneri* triggered macrophage death. They classified this death as apoptosis because they observed typical morphological changes of apoptosis in the cell including, cell rounding, shrinkage, blebbing and lysis during death [17]. Furthermore, Hilbi found that *S. flexneri* bound directly to caspase-1 during the process. The invasion plasmid antigen B (ipaB) present in the cytoplasm bunds to caspase-1 [interleukin 1-β (IL-1β) converting enzyme], leading to its activation [18, 19]. Caspase-1 activity is pivotal for the induction of *Shigella*-induced macrophage apoptosis. Hersh et al. also observed caspase-mediated ‘apoptosis’ of macrophages in 1999, following microinjection with the secreted protein SipB from *Salmonella* spp. However, caspase-1 knockout macrophages were not susceptible to *Salmonella*-induced cytotoxicity [20]. Both Chen and Hersh observed that this so-called “apoptosis” would promote secretion of IL-1β which not only initiates the process of inflammation, but also cell death which similar to necrosis [17, 20].

It was not until 2001, when Brennan used the term ‘Pyroptosis’ to describe this combination of apoptosis and necrosis [21]. “Pyro” in Greek means fire, and “Ptosis” means falling, reflecting the fact that the essence of cell death is accompanied by inflammation. Pyroptosis remains a highly conserved process that might exist in all vertebrates as a mechanism to resist pathogens and promote the immune system. Studies have provided evidence for pyroptosis in terrestrial animals, for example, mice (*Mus musculus*), humans (*Homo sapiens*) [22, 23], and also in aquatic species, such as, large webbed bombina (*Bombina maxima*) [24], sea bream (*Sparus aurata*) [25], Atlantic salmon (*Salmo salar*) [26], and zebrafish (*Danio rerio*) [27, 28].

## Morphological changes of pyroptosis

**Fig. 1:**
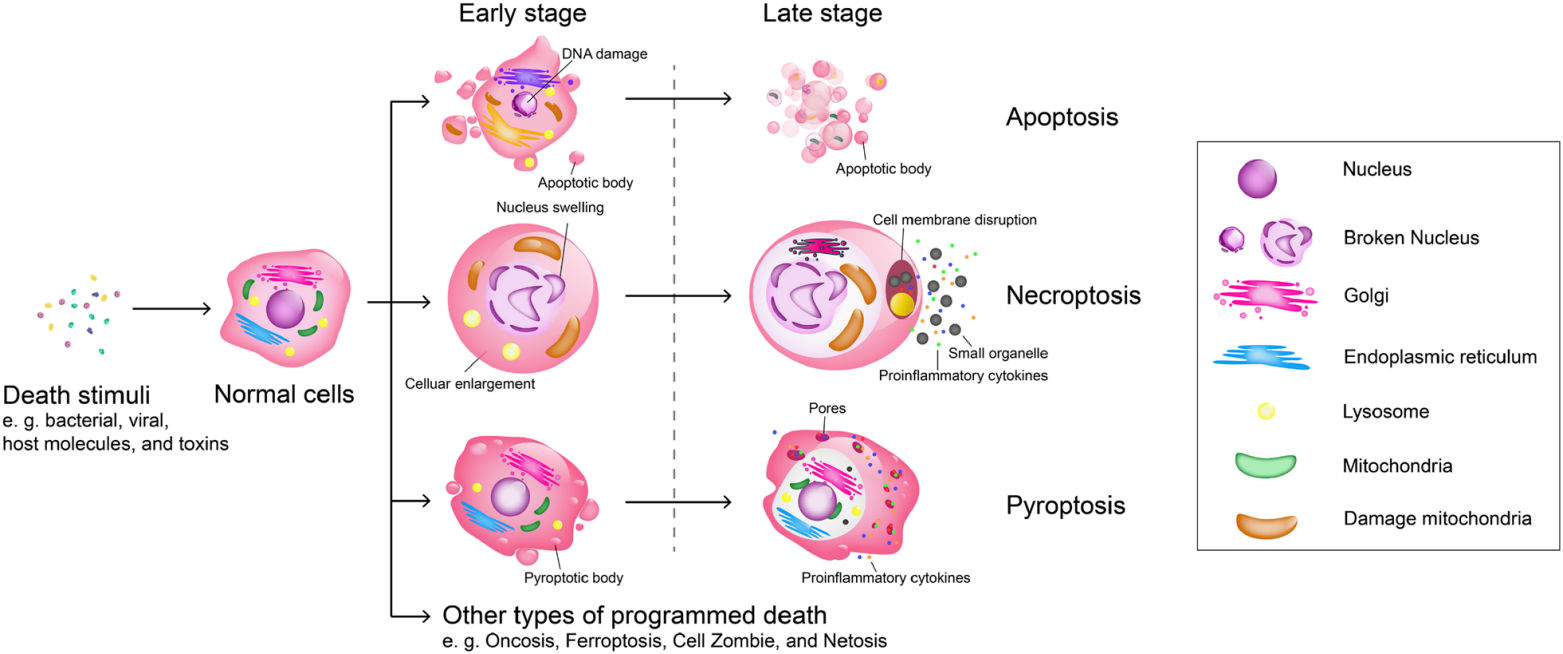
Morphological changes of programmed cell death [2, 29]. Cells initiate different pathways of cell death upon overstimulation. The morphological changes of apoptosis include chromatin condensation, cell membrane fragmentation, and the formation of apoptotic bodies. The morphological characteristics of necroptosis are mainly characterized by swelling and rounding of the cells, swelling of the cytoplasm, enlargement of organelles, and disruption of the cell membrane. Whereas the morphological changes of pyroptosis are manifested by the formation of small pores on the cell membrane and the release of inflammatory cytokines

Pyroptosis is a form of cell death which occurs in phagocytes (macrophages, monocytes, and dendritic cells (DCs)) [29], and comprises mainly of two stages: early and late. In the early stage, a large number of vesicles are produced on the cell membrane, called pyroptotic bodies [30]. The shape and size of pyroptotic bodies are similar to apoptotic bodies. In the late stage, a large number of pores (10–14 nm in diameter) appear on the cell membrane and induce the rupture of the cell membrane (Fig. 1) [30]. Moreover, intracellular substances such as IL-1β, IL-18, and lactate dehydrogenase are released through the pores, resulting in an inflammatory response surrounding the dead cells [31]. It is difficult to separate pyroptosis from apoptosis and necroptosis by morphological changes, as only small distinctions are apparent in histomorphology. The greatest similarity between pyroptosis and apoptosis is the production of pyroptotic and apoptotic bodies. The greatest similarity between pyroptosis and necroptosis is the release of cytoplasmic content and proinflammatory factors. In contrast, apoptosis does not result in inflammation, and necroptosis does not result in the formation of vesicles. Indeed, apoptosis appears more like an aging process, with the increase of intracytoplasmic garbage, fragmentation of nuclear material, atrophy of cells, and the eventual formation of apoptotic bodies which are exfoliated or engulfed by surrounding cells (Fig. 1). In addition, necroptosis can be compared to a bursting balloon, where cell death is initiated by receptors such as tumour necrosis factor receptor 1 which requires receptor-interacting protein 1 (RIP1) (also known as RIPK1) and RIP3 (also known as RIPK3), resulting in cell detachment, swelling, disintegration of mitochondrial, lysosomal and plasma membranes and the ultimate lysing of the cell [32]. The cytoplasm of the dead cells, including the organelles is released (Fig. 1). In comparison, pyroptosis is more comparable to a filter, which produces a large number of holes (pyroptotic body) on the surface of the cell membrane, resulting in the release of a large number of small cellular components (proinflammatory cytokines) into the cytoplasm, while cellular components larger than the holes (macromolecular proteins, organelles) are left inside (Fig. 1) [30].

## How to accomplish pyroptosis through caspase-1 induced canonical inflammatory pathway

Caspase-1-activating platforms called “inflammasomes”, which are multi-protein signaling complexes that assemble in response to detection of cellular disruption and noxious stimuli such as bacterial, viral and host molecules, toxins, pollutants, crystals, UV radiation etc, and initiate inflammatory immune responses. The formation of the inflammasomes marks that caspase-1 will be activated, triggers a series of cascade reactions and accomplish pyroptosis.

Currently, the known inflammasome receptors include three gene families: Nod-like receptors (NLRs) [33], AIM2-like receptors (ALRs) [34], and the tripartite motif family (TRIM) of which only pyrin is an inflammasome [35]. In addition, the adaptor protein-ASC (apoptosis-associated speck-like protein) is expressed in epithelial tissues and immune cells such as neutrophils and macrophages and consists of two death-fold domains: one pyrin domain (PYD) and one caspase activation and recruitment domain (CARD) [36]. While the exact composition of the inflammasome is dependent on the initiating compound [37, 38] both the receptor family and the ASC are critical components of the inflammasome that are involved in the recruitment and activation of the caspase-1 precursor, procaspase-1 [39, 40].

**Fig. 2:**
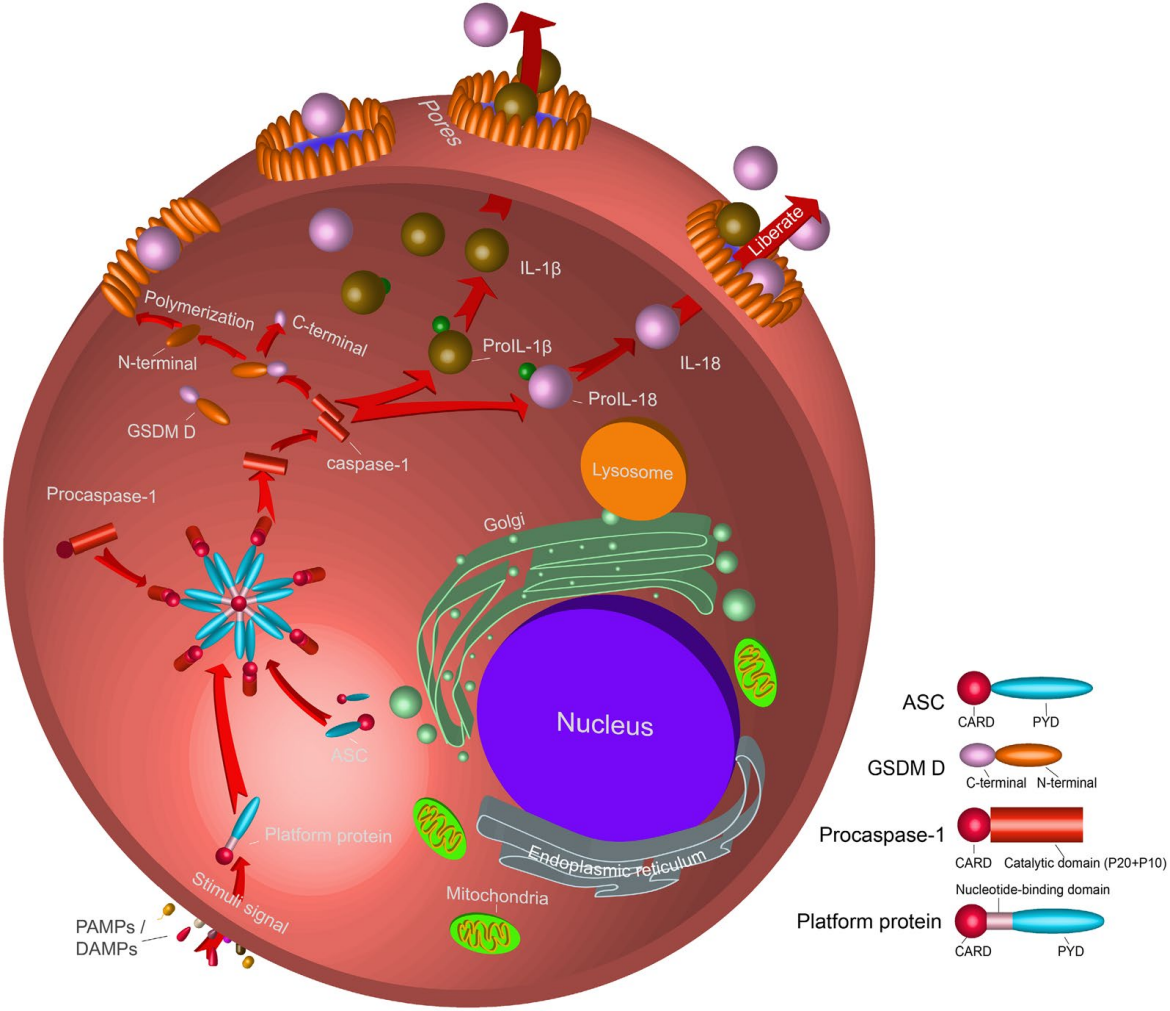
Regulation of caspase-1 mediated canonical inflammatory pathway. Platform protein, which mainly belong to NLRs, ALRs, and TRIM families. In the inflammasome, the Asp-x cleavage site between the p20 and p10 subunits of caspase-1 was hydrolyzed, followed by the CARD. The cleaved p20 and p10 subunits are polymerized in the form of tetramers to become activated caspase-1

In the initial process of pyroptosis the inflammasome activates caspase-1 through a Nod-like receptor (NLRP1, 3, 6, 7, 12, NLRC4), AIM2, or pyrin, all of which contain a CARD or pyrin domain (PYD) [41, 42]. The CARD domain is able to recruit and bind procaspase-1 through the CARD–CARD and PYD–PYD interactions [36, 43]. The recruitment of procaspase-1 into the ASC via the CARD–CARD interactions which results in its cleavage into its N-terminal pro-domain and C-terminal catalytic and interaction domains; all caspases contain two essential caspase catalytic domains: the p10 subunit and the p20 subunit, the latter containing the key catalytic residues cysteine and histidine. These two cleaved C-catalytic domains integrate with each other and formed mature/activated caspase-1 (Fig. 2) [44].

The mature/activated caspase-1 induces the formation of the pores in the cell membranes, on the other it could mature the intracellular inflammatory cytokines and assist in ecliciting the inflammatory response. During this process, caspase-1 has been shown to hydrolyze another protein named Gasdermin (GSDM) D in mouse and human cells. GSDM D belongs to the Gasdermin family that includes GSDM A, B, C, D, E, and DFNB59 [45]. Also, GSDM D is a protein mainly expressed in immune cells [45] and was discovered to form a pore and act as the effector for pyroptosis [46]. The GSDM D protein polypeptide chain consists of a total of approximately 480 amino acids and has two major domains: the N-terminal pore-forming domain (PFD) and the C-terminal repressor domain (RD) which are linked by a linker loop [5]. The PFD plays a crucial role in the formation of pyroptosis, and the main function of the RD is to inhibit the activity of the GSDMD-N domain in normal cells (Fig. 2) [47].

When caspase-1 binds to GSDM D, it will cleave the linker loop between PFD and RD, and the cleaved site is located in D_276_↓G_277_. Cleaved proteins result in the separation of two domains, and PFD will play an important role in the following process (Fig. 2). Ding et al. [47] found that PFD appeared as oligomers and generate an oligomeric ring after cleaved, which diameters in 10–14 nm. Robin extracted the ring and determine the protein size of about ~ 720 kDa on a blue native-page gel [48]. Based on the results, we speculate that 24 PFDs can be aggregated to form a protein pore. The resulting pores appear on the cell membrane, resulting in changes in membrane permeability, like membrane breakage. The remaining question is how the pores appear on the cell membrane?

Polyprotein pore has the greatest affinity for lipid species [47]. Rogers et al. generated a model of DFNA5 (GSDM E, homology to GSDM D) and conjecture that the interior of the multimeric protein harbours a membrane targeting motif, relies on this motif, which locates the multimeric protein at the cell membrane, resulting in numerous small pores in the cell membrane [49, 50]. Eventually, substances smaller than the pores, such as Na^+^, water, cytoplasmic proteins, and proinflammatory factors, will flow out of the pores, while larger organelles, such as ribosomes, will be blocked in the cells. Eventually, the cells die and a series of inflammatory reactions are initiated around the cells [51] (Fig. 2).

## Whether the caspase-1-mediated pathway is the only channel that regulates pyroptosis?

The caspase-1 mediated pathway has been considered to be the only pathway to mediate pyroptosis for many years. In 2011, Kayagaki observed that caspase-11 is critical for IL-1β production and pyroptosis in macrophages infected with *Escherichia coli*, *Citrobacter rodentium* and *Vibrio cholerae*, which revealed a different regulatory pathway for pyroptosis [52]. However, it spent a long time from the identification of caspase-11 to verify its unique pathway. In the mid-1990s, scholars succeeded in cultivating knockout caspase-1 (CASP1^−/−^) and caspase-11 (CASP11^−/−^) mice. Li et al. [53] found that CASP1^−/−^ mice were resistant to endotoxic shock induced by LPS, and Wang et al. [54] reported the same resistance in CASP11^−/−^ mice. During the development of CASP1^−/−^ and CASP11^−/−^ mice, it was observed that mutant mice were resistant to LPS infection, while wild-type mice developed significant lethal sepsis. Due to the similarity of the results of these studies, the current belief at that time and for the next decade was that these two genes were closely related to inflammation and played a role in regulating the same pathway.

Later studies have however since revealed that caspase-1 and caspase-11 play different roles in the inflammatory response [52]. In 2011, Kayagaki et al. observed that CASP11^−/−^ mice exhibited defects in IL-1β production in response to *E. coli*, *C. rodentium* or *V. cholerae*, but secreted IL-1β normally in response to monosodium urate and adenosine triphosphate (ATP). Based on this result, these authors speculated that caspase-11 mediated an unusual proinflammatory pathway. Their study demonstrated a loss of caspase-11 rather than caspase-1 protected mice following a lethal dose of LPS, and that caspase-11 does not require NLRP3 and ASC in processing cell death. These data highlight a unique proinflammatory role for caspase-11 to clinically significant bacterial infections. In addition, the authors suspected that the pathway was induced by LPS specific stimulation to induce cell death [52]. More studies have also shown that the caspase-11-mediated pyroptosis has strong specificity for LPS. G^−^ bacteria such as *V. cholerae*, ΔFlag *Salmonella*, *E. coli*, enterohaemorrhagic *E. coli*, *Legionella pneumophila*, *C. rodentium* could all induce activation of the caspase-11 in macrophages [55, 56], but G^+^ bacteria did not demonstrate similar results [56]. In addition, LPS-mutant *E. coli* failed to activate caspase-11 [8]. Since then, the caspase-11-mediated pathway in pyroptosis has been named as the non-canonical inflammatory pathway to highlight its specific ability to recognize the LPS on G^−^ bacteria surface.

### Relationship between caspase-1 and caspase-11

**Fig. 3:**
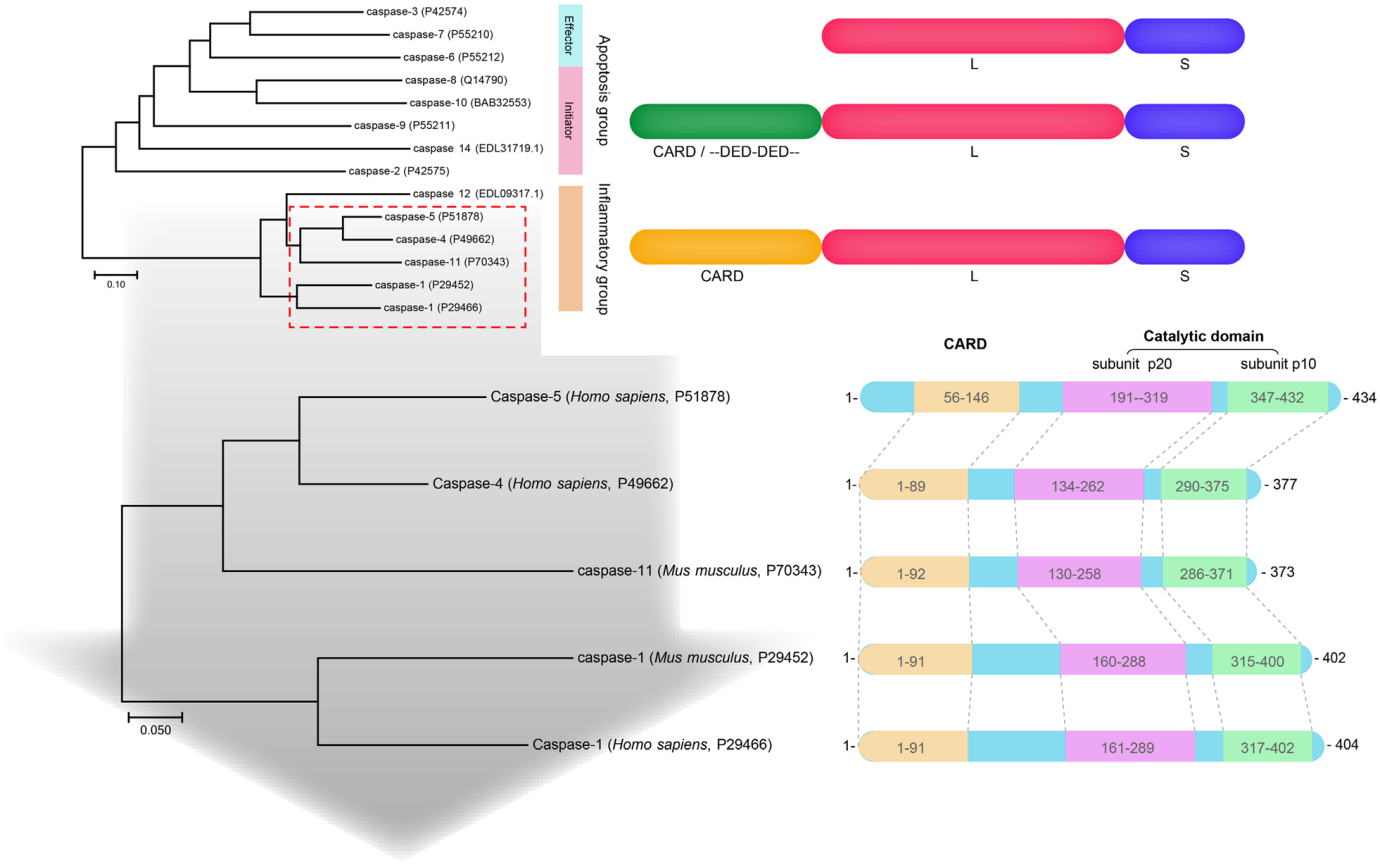
Caspase family homology comparison (Source: https://www.ncbi.nlm.nih.gov/). A phylogenetic tree was constructed by the N-J method to compare the homology among members of the Caspase family. CARD, Caspase activation and recruitment domains; L + S, catalytic domain, L as p20 subunit an d S as p10 subunit. Protein domains were predicted by interpro (www.ebi.ac.uk/interpro/scan)

In 1996, Chen [17] reported an enzyme that has the ability to activate proIL-1β and proIL-18 which they named IL-1β converting enzyme (ICE). However, subsequent studies found the greater significance of ICE is to “awaken” and “execute” cell death [53, 57, 58]. IL-1β converting enzyme and similar proteins were established as a new group, caspase family, and ICE was renamed caspase-1. The caspase family consists of 15 homologous proteins, and has certain species specificity [59]. According to phylogenetic analysis, the caspase family can be divided into an apoptosis subfamily represented by caspase-3 and an inflammatory subfamily represented by caspase-1 (Fig. 3).

Caspase-1 and caspase-4, -5/-11 belong to the proinflammatory caspase subfamily, and their highly homologous sequences imply their functional similarity [59]. Caspase-11 has been cloned and identified in mice [60] and caspase-4/-5 has been identified in humans, both of which have showed up to 60% homology in amino acid sequence [61, 62] (Fig. 3). In addition, Shi et al. [63] demonstrated that caspase-4/-5 functionally similar to murine caspase-11 could induce human monocytes, epithelial cells, and keratinocytes pyroptosis. The similarity between caspase-1 and caspase-11 (-4, -5) may be the reason why they can play a similar role in pyroptosis, however, the heterogeneity between the sequences of caspase-1 and caspase-11 may be responsible for their mediation of two different pathways [64, 65].

### The different structure of caspase-11 and caspase-1 indicates different function

**Fig. 4:**
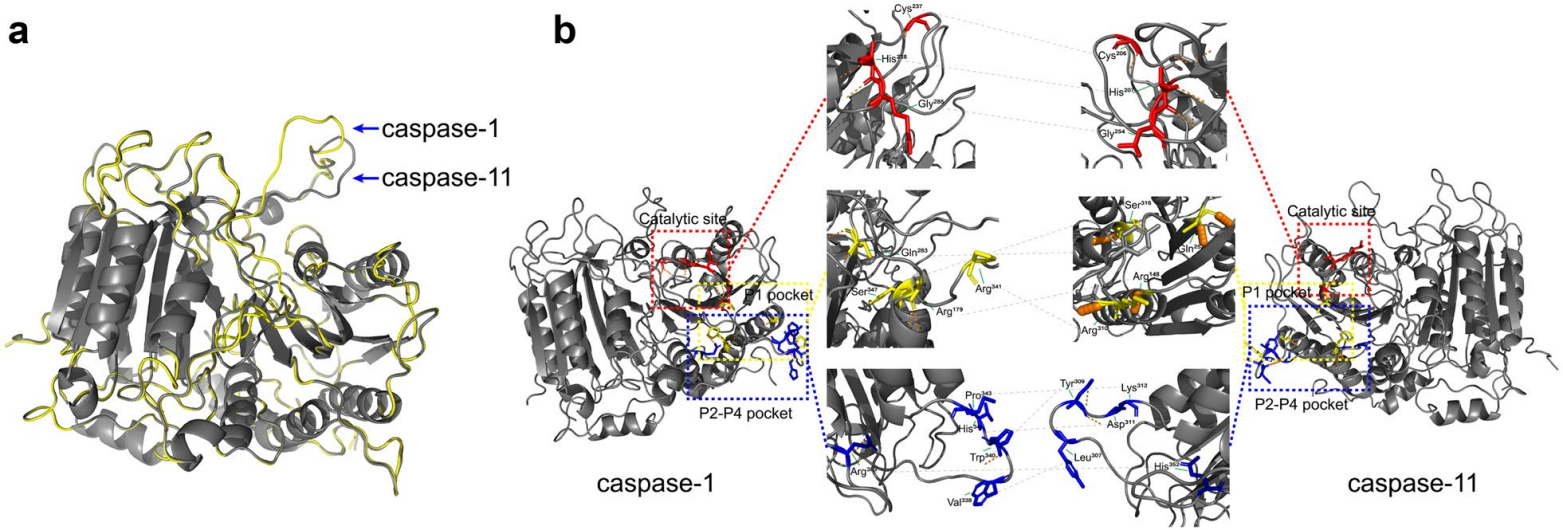
Comparison of the tertiary structure of caspase-1 and caspase-11. **a** Align of molecular structure of caspase-1 and caspase-11; **b** Comparison of active sites of caspase-1 and caspase-11. Red represents the catalytic sites; Yellow represents the P1 pocket, and blue represents the P2–P4 pockets. The tertiary structure is drawn by PyMOL, Accession Number: caspase-1: P29452 [69]; caspase-11: P70343 [60]

The *caspase*-*11* cDNA has a full length of 1350 bp and contains an open reading frame (ORF) encoding 373 amino acids. The initiation codon is located at 35–37 nucleotides and the stop codon is located at 1154–1156 nucleotides of the cDNA sequence [60]. There are 39 Poly (A) signals (AATAAA) at the end of the non-coding region of the cDNA, and the predicted molecular weight of the encoded caspase-11 protein is 43 kDa. As with caspase-1, caspase-11 is composed of an N-terminal CARD domain and a C-terminal caspase catalytic (subunit p20 + p10) domain. In addition, between each domain, there is a conserved hydrolysis site. In caspase-1, cleavage between the subunit p20 and subunit p10 is dependent on the aspartate residue (Asp^−^). Similarly, the protein structure of caspase-11 was predicted and it was found that the cleavage site of p20 and p10 subunit is similar to caspase-1 and highly conserved [60]. Moreover, the main catalytic sites of caspase-1 are His^237^, Gly^238^ of p20 subunit, and Cys^285^ of p10 subunit, which is conserved in caspase-11 (His^206^, Gly^207^ and Cys^254^) [66, 67]. In addition, the partial sequence of the P1 pocket of caspase-1 where the catalytic site is located (Arg^179^, Gln^283^, Arg^341^, and Ser^347^) is also conserved in caspase-11 (Arg^148^, Gln^252^, Arg^310^, and Ser^316^) (Fig. 4). The results demonstrate that the function of caspase-11 may be the same as that of caspase-1, both of which perform enzymolysis through its P1 active group and choose the Asp site of the substrate for enzymolysis.

However, some functional loci may be responsible for their functional differences. Compared to caspase-1, the hydrolysis site between p20 and CARD is not conserved in caspase-11 [66–68]. In caspase-1, the cleavage between the p20 and p10 subunits is dependent on the Asp^−^ at the p10 position. However, there are two adjacent Asp- residues (Asp^59^ and Asp^80^) in caspase-11, which may be potential hydrolysis sites at the N-terminus when processing p20, and procaspase-11 is hydrolyzed here to produce a 20 k Da subunit. In addition, the sites that constitute P2–P4 pocket in caspase-1 (Val^338^, Trp^340^, His^342^, Pro^343^, Arg^383^, and Gln^385^) are different in caspase-11 (Leu^307^, Tyr^309^, Asp^311^, Lys^312^, and His^352^), only Gln^354^ is conserved with caspase-1 [64]. The P2–P4 pocket is the binding group of caspase-11, suggesting that the difference in the substrates used may be the main reason for mediating the two different pathways (Fig. 4).

## How does caspase-11 regulate non-canonical inflammatory pathways?

While the signaling pathways and molecular mechanisms of non-canonical inflammatory pathways need to be further characterized, an almost complete pathway has been described in the present study. The activation of the canonical inflammatory pathway often requires the formation of inflammasomes, although Kayagaki et al. [52] found that caspase-11 does not seem to require NLRP3 and ASC to induce pyroptosis formation, suggesting that the caspase-11-mediated non-canonical inflammatory pathway may complete the death process through another mechanism. Researchers have demonstrated there are two stages of non-canonical inflammatory pathways, initiation and excitation [8]. In the initial stages, G^−^ bacteria activate the transcriptional expression of intracellular genes, generating a large amount of procaspase-11 and inflammation-related inflammatory cytokines. Once procaspase-11 is synthesized, non-canonical inflammasome is activated by cytosolic recognition of intracellular LPS derived from bacteria that have escaped phagolysosomes, and trigger the cell death. This pathway is independent of Toll-like receptor 4 (TLR4), the well-known extracellular receptor for LPS, but instead depends on the inflammatory protease, caspase-11.

### The process of producing procaspase-11 and other inflammatory factors

When G^−^ bacteria invade the body, the immune system is activated, and the initial stages of pyroptosis activate. Innate immune cells, especially macrophages, can rapidly recognize the evolutionarily conserved structures on pathogens, pathogen-associated molecular patterns (PAMPs) of invading pathogens through a limited number of pattern recognition receptors (PRRs), such as scavenger receptors and the family of Toll-like receptors (TLRs) which have been studied most extensively. Pattern recognition receptors present at the cell surface or intracellularly signal the presence of infection and elicit proinflammatory and antimicrobial responses by activating a intracellular signaling pathways, including adaptor molecules, kinases, and transcription factors [70]. PRR-induced signal transduction pathways ultimately result in the transcription and translation of genes, including *caspase*-*11* and *IL*-*1β*, for the non-canonical inflammatory response, including *NLRP3*, *caspase*-*1* and *IL*-*18* [71, 72].

**Fig. 5:**
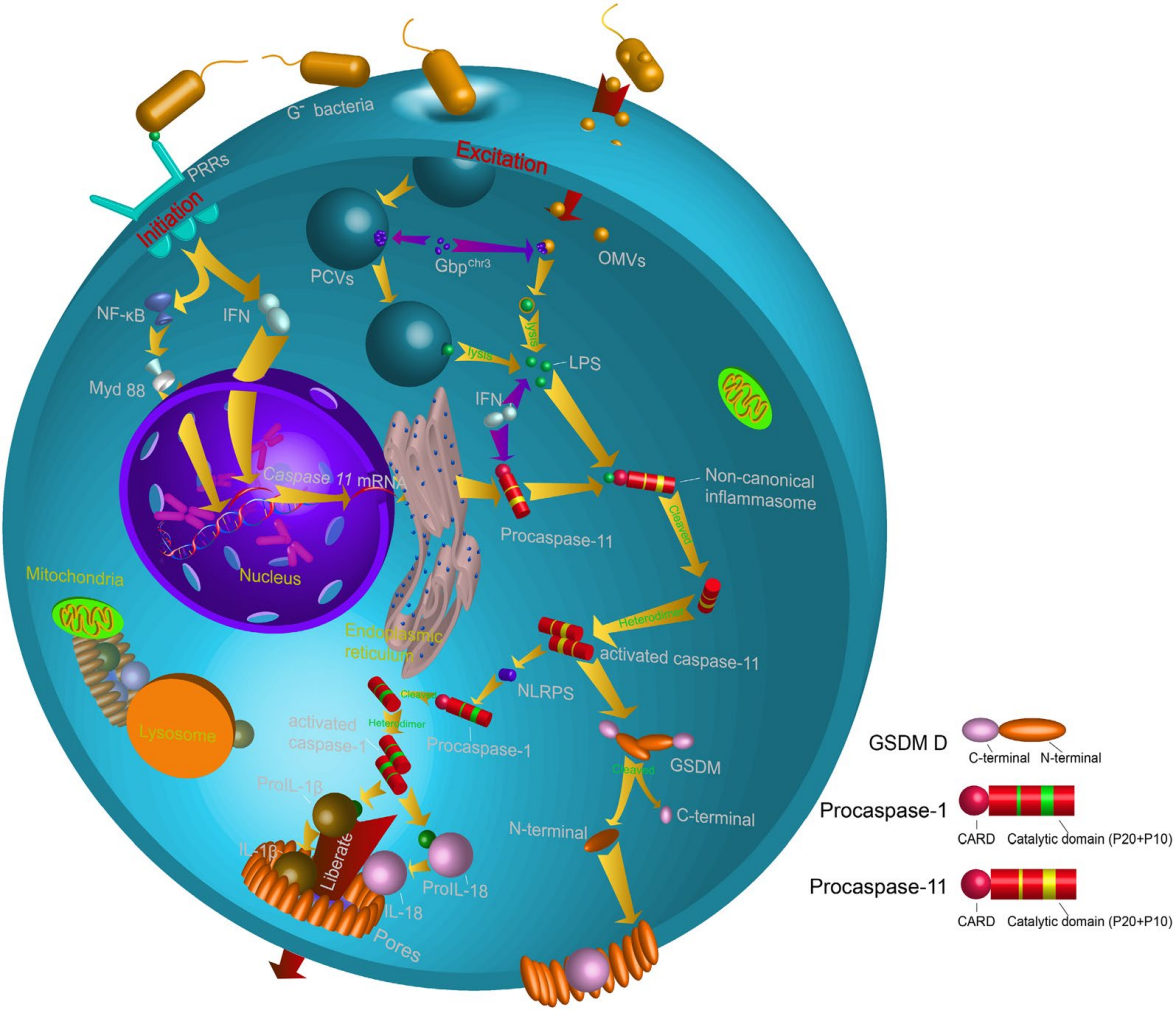
The regulation of caspase-11 involved non-canonical inflammation. The non-canonical inflammatory pathway was divided into two parts: initiation and excitation, both of which are activated by LPS. The difference is that the initiation process depends on the recognition of LPS by receptors on the cell membrane, and the excitation process requires LPS to enter the cell

Is there a mechanism to expand and accelerate the production of procaspase-11 or other proinflammatory factors? Recent studies show that most of the genes in pyroptosis are constitutively expressed. For example, during the transcription of *caspase*-*11*, the 5′-terminal of the *caspase*-*11* contains multiple *NF*-*κB* binding sites and a *STAT* binding site. TLR receptors or IFNs sequentially activate *NF*-*κB* and *STAT* transcription, and a large amount of procaspase-11 is produced during the progress. In addition, TLR receptors or IFNs cascade amplify the expression of these inflammatory genes. For example, TLR 2 promotes the expression of *caspase*-*11* by activating the NF-*κ*B-transduction of the Myd88 [8, 73]. TLR 3 also promotes the expression of *caspase*-*11* by inducing the production of IFN-α/β (IFN-I) [56]. After transcription and translation, a large amount of procaspase-11 and proIL-1β, proIL-18 were generated and distributed in the cytoplasm (Fig. 5).

### Non-canonical inflammasome formation and procaspase-11 maturation

Under certain conditions, inflammation is induced by a canonical inflammatory pathway and recognized and activated by cell membrane surface receptors like TLR 4 [74]. In contrast, non-canonical inflammasomes, i.e. the complex of procaspase-11 and LPS, are activated in cells. LPS can activate the transcription of *caspase*-*11* through TLR or other receptors. So, does LPS also cleave and generate mature caspase-11 by signal transduction of a cell surface receptor? Recent studies have discovered that the host is capable of TLR4-independent recognition of LPS in the cytosol [75]. In addition, Shi et al. found that not only murine caspase-11 but also human caspase-4 and -5 do not employ an upstream signaling cascade to detect LPS, but instead sense LPS directly [63]. LPS binds with procaspase-11, initiate procaspase-11 oligomerization and form mature/activated caspase-11. However, binding-deficient CARD-domain point mutants in procaspase-11 did not respond to LPS with oligomerization or activation and failed to induce pyroptosis upon LPS electroporation or bacterial infections, indicating that the binding site of LPS is located on the CARD domain of procaspase-11 [63]. So, how does LPS enter the cell to bind to procaspase-11?

Vanaja et al. [76] reveal a fundamental mechanism that delivers LPS into the cytosol to alert the immune system. Outer membrane vesicles (OMVs) are a naturally secreted product of G^−^ bacteria, and LPS is one of the most abundant components of OMVs [77]. The recent study showed that the size, shape and membrane composition of OMVs are similar to that of extracellular vesicles formed by eukaryotic cells [78], and Vanaja and colleagues [76] found OMVs secreted by G^−^ bacteria function as a vehicle to deliver LPS into the cytosol from early endocytic compartments and lead to pyroptosis. However, Etienne et al. [79] found that host cells could consume G^−^ bacteria through pathogen-containing vacuoles (PCVs) formed by eukaryotic cell membrane, suggesting that LPS may enter the cytoplasm through multiple pathways.

The next step is to understand how the host cells detect the swallowed vesicles. There are 11 genes encoding guanylate binding protein (Gbp) in murine cells, and GBP^chr3^ (GBP encoded on chromosome 3) plays an important role in the lysis of the vacuoles of G^−^ bacteria [80]. Initially, Pilla et al. [80] discovered that Gbp^chr3^ play a role in the detection of cytoplasmic LPS and the subsequent activation of the noncanonical inflammasome leading to pyroptosis. Another study observed large amounts of GBP^chr3^ attached on the vesicle membrane, resulting in the vesicle lysis, and eventually, the bacteria released into the cytoplasm (Fig. 5) [79]. In addition, a process negatively regulated by a subclass of IRG proteins called IRGMs may play an important role in the recognition of non-self vesicles by GBP. The paralogous IRGM proteins Irgm1 and Irgm3 fail to robustly associate with non-self PCVs but instead reside on self organelles including lipid droplets (LDs). Whereas IRGM-positive LDs are guarded against the stable association with GBPs [81]. Furthermore, Gbp1 and Gbp2 could successful localize to IRGM^−/−^ LDs, and IRGM^−/−^ LDs further be delivered to autophagosomes for degradation, which evident that IRGM proteins act as ‘‘guards’’ that prevent lysis of non-self vesicles [81].

Cierra et al. discovered that TRIF-dependent type I Interferon (IFN-I) and its related pathway is necessary for the activation of caspase-11. When LPS enters into the macrophages the cells modulate IFN-I signaling-induced gene expression to assist procaspase-11 to recognize the intracellular LPS, and ultimately activate caspase-11 [82]. Since then, the CARD domain of caspase-11 interacts with LPS to form the procaspase-11-LPS complex, also known as non-canonical inflammasome [75]. Procaspase-11 is cleaved and activated as caspase-11 in the non-canonical inflammasome.

### Caspase-11 mediated proinflammatory response

The most important feature of pyroptosis is the release of proinflammatory cytokines from cells and the induction of inflammation. In canonical inflammatory pathways, caspase-1 could directly cleave proIL-1β and proIL-18 and produce mature/activated IL-1β and IL-18. However, Kayagaki found that CASP11^−/−^ macrophages did not affect the maturation of IL-1β and IL-18 following G^−^ bacterial injection, while the mature IL-1β and IL-18 in CASP1^−/−^/CASP11^−/−^ macrophages were significantly reduced. At the same time, the expression of caspase-1 was observed in G^−^ bacteria-injected macrophages. These results indicate that caspase-11 itself may not have the ability to cleave proIL-1β and proIL-18 and that maturation still requires the help of caspase-1 (Fig. 5) [8, 52]. Kayagaki [52] then discovered that caspase-11 needs to induce NLRP3 activation through an unknown pathway to promote the activation of procaspase-1, and that activated caspase-1, in turn, induces the activation and maturation of proIL-1β and proIL-18 (Fig. 5).

Recent studies have found that GSDM D plays an important role in both the caspase-1 mediated canonical inflammatory pathway and the caspase-11 mediated non-canonical inflammatory pathway [50, 83]. When caspase-11 is activated, it induces the downstream substrate GSDM D to cleave into the N terminal and C terminal domains, with the N terminal domain on the cell membrane. Cleavage removes the C-terminal fragment (GSDMD-CT). The N-terminal oligomerizes in membranes to form pores that triggers pyroptosis [49]. With caspase-1 cleaving the interleukin precursor (proIL)-1β and proIL-18 in the cell and releasing mature/activated IL-1β and IL-18. Mature/activated IL-1β and IL-18 along with other cytoplasmic components are released from the pores (Fig. 5) [84]. The release of these proinflammatory factors would further lead to cellular inflammation.

## Conclusion

Compared with G^+^ bacteria, G^−^ bacteria have a layer of LPS on the cell wall, which is also called “endotoxin”. The presence of endotoxins in the blood is called endotoxemia, which leads to septic shock [85]. Moreover, LPS is thought to cause autoimmune-based host responses, such as multiple sclerosis [86]. Non-canonical inflammatory pathways of pyroptosis play an important role in inhibiting the proliferation of G^−^ bacteria and scavenging bacteria, and could effectively prevent the massive deposition of LPS. Therefore, the exploitation of drugs that stimulate the expression of caspase-11 and activate non-canonical inflammatory pathways will be beneficial for the treatment of a large number of bacterial diseases.

In this paper, we summarize the current research of non-canonical inflammatory pathways-induced pyroptosis, which is related to the host cell response to G^−^ bacteria. By carefully studying the molecular processes that occur in cells in non-classical inflammatory pathways and paying attention to the results of pyroptosis that affect the development of inflammation and immune responses, we will better characterize new pathways of cell death and further understand the interactions between host and G^−^ bacteria. However, there is still gaps in the knowledge on the study of non-canonical inflammatory pathways. First, the non-canonical inflammatory pathways are only studied in humans and mice, and it is unclear whether other species have the same mechanism. For example, do zebrafish recognize intracellular LPS by NOD1 instead of caspase-11 (-4,-5) homologs [87]? In addition, the mechanism of non-canonical inflammatory pathways is not complete, and questions exist as to whether OMVs and PCVs coexist? What is the inhibition mechanism of caspase-11 (only few studies indicated that NleF and could inhibit caspase-11 activation [88, 89])? The study of non-canonical inflammatory pathways must be a process full of exploration of the unknown.

## Abbreviations

Caspase: cysteinyl aspartate-specific proteinase
Procaspase: caspase precursor
LPS: lipopolysaccharide
G^−^: gram-negative
G^+^: gram-positive
IL: interleukin
proIL: IL precursor
ICE: IL-1β converting enzyme
CASP^−/−^: knocked out caspase
Asp^−^: aspartic acid residue
TLR: Toll-like receptors
PRRs: pattern recognition receptors
ALRs: AIM2-like receptors
TRIM: triplet motif family
NLRP3: NLR family pyrin domain containing 3
NF-Κb: Nuclear factor-kappa B complex subunits
STAT: signal transducers and activators of transcription
IFNs: interferons
Myd88: myeloid differentiation factor 88
GBP: guanylate-binding protein
GBPchr3: GBP encoded on chromosome 3
CARD: caspase recruitment domain-containing
GSDM D: Gasdermin D
DFNA 5: Gasdermin E
